# Comprehensive Analysis of Necroptosis in Pancreatic Cancer for Appealing its Implications in Prognosis, Immunotherapy, and Chemotherapy Responses

**DOI:** 10.3389/fphar.2022.862502

**Published:** 2022-05-18

**Authors:** Kun Fang, De-Sheng Tang, Chang-Sheng Yan, Jiamin Ma, Long Cheng, Yilong Li, Gang Wang

**Affiliations:** ^1^ Department of Pancreatic and Biliary Surgery, First Affiliated Hospital of Harbin Medical University, Harbin, China; ^2^ Key Laboratory of Hepatosplenic Surgery, First Affiliated Hospital of Harbin Medical University, Harbin, China

**Keywords:** necroptosis, pancreatic cancer, prognosis, immunogenicity, immunotherapy, chemosensitivity

## Abstract

**Objective:** Necroptosis represents a new target for cancer immunotherapy and is considered a form of cell death that overcomes apoptosis resistance and enhances tumor immunogenicity. Herein, we aimed to determine necroptosis subtypes and investigate the roles of necroptosis in pancreatic cancer therapy.

**Methods:** Based on the expression of prognostic necroptosis genes in pancreatic cancer samples from TCGA and ICGC cohorts, a consensus clustering approach was implemented for robustly identifying necroptosis subtypes. Immunogenic features were evaluated according to immune cell infiltrations, immune checkpoints, HLA molecules, and cancer–immunity cycle. The sensitivity to chemotherapy agents was estimated using the pRRophetic package. A necroptosis-relevant risk model was developed with a multivariate Cox regression analysis.

**Results:** Five necroptosis subtypes were determined for pancreatic cancer (C1∼C5) with diverse prognosis, immunogenic features, and chemosensitivity. In particular, C4 and C5 presented favorable prognosis and weakened immunogenicity; C2 had high immunogenicity; C1 had undesirable prognosis and high genetic mutations. C5 was the most sensitive to known chemotherapy agents (cisplatin, gemcitabine, docetaxel, and paclitaxel), while C4 displayed resistance to aforementioned agents. The necroptosis-relevant risk model could accurately predict prognosis, immunogenicity, and chemosensitivity.

**Conclusion:** Our findings provided a conceptual framework for comprehending necroptosis in pancreatic cancer biology. Future work is required for evaluating its relevance in the design of combined therapeutic regimens and guiding the best choice for immuno- and chemotherapy.

## Introduction

Pancreatic cancer is one of the most lethal human cancers with an undesirable five-year survival rate < 10% (Yu et al., 2021). In 2018, there were 458,918 newly diagnosed pancreatic cancer cases and 432,242 death cases worldwide (Rawla et al., 2019). Surgical resection is currently the only therapeutic option with curative potential (Zhu et al., 2019a). Nevertheless, when diagnosed, about 80–85% patients have developed an unresectable or metastatic state (Tao et al., 2021). Even for the minority of patients who have the opportunity to receive surgical resection, only 20% can survive for 5 years (Zhu et al., 2020). Adjuvant chemotherapy with FOLFIRINOX (fluorouracil, irinotecan, leucovorin, and oxaliplatin) as a standard treatment option can prolong patients’ long-term outcomes, with a median overall survival of 54.4 months (Park W. et al., 2021). Nevertheless, intrinsic and acquired resistance to chemotherapy is still a thorny issue in pancreatic cancer therapy (Zhu et al., 2019b). At present, a few clinical trials are ongoing to evaluate the efficacy of immunotherapy in pancreatic cancer (O'Hara et al., 2021; Pang et al., 2021; Zhu et al., 2021). Regrettably, none of these trials fail to show satisfying outcomes (Schizas et al., 2020). Hence, it is urgently required to design novel therapeutic regimens specifically targeting pancreatic cancer biology.

Necroptosis is a form of regulated necrotic cell death mainly mediated by receptor-interacting protein kinase 1 (RIPK1), RIPK3, and mixed lineage kinase domain-like (MLKL) protein (Gong et al., 2019). Necroptosis has become a new target for cancer immunotherapy because it is considered a form of cell death overcoming apoptosis resistance that enhances tumor immunogenicity, which is particularly important for the treatment of immune-desert tumors (Tang et al., 2020b). For instance, RIPK3 activation-triggered de-inhibition of tripartite motif protein 28 (TRIM28) in tumor cells results in increased immunostimulatory cytokine production within the tumor microenvironment and thus contributes to robust cytotoxic antitumor immunity (Park H. H. et al., 2021). Previous studies have uncovered the significance of necroptosis in pancreatic cancer. For instance, necroptosis facilitates pancreatic cancer cell migration and invasion through releasing CXCL5 (Ando et al., 2020). The aurora kinase inhibitor CCT137690 triggers necroptosis in pancreatic cancer cells through RIPK1, RIPK3, and MLKL and thus suppresses tumor growth (Xie et al., 2017). Necroptosis-induced CXCL1 and Mincle signaling facilitate macrophage-mediated adaptive immune inhibition and thus enhance pancreatic cancer progression (Seifert et al., 2016). In-depth understanding of necroptosis is crucial for immune surveillance and treatment management.

In our study, we clustered five robust necroptosis subtypes of pancreatic cancer, following the consensus clustering approach based on prognostic necroptosis genes. The five necroptosis subtypes displayed diverse prognosis, immunogenic features, genomic mutations, and chemosensitivity, providing a reference for combined therapeutic regimens and guiding the best choice of patients for immuno- and chemotherapy. Moreover, we developed a necroptosis-relevant risk model for reflecting necroptosis subtypes in clinical practice.

## Materials and Methods

### Collection and Integration of Transcriptomic Data on Pancreatic Cancer

This study retrospectively collected transcriptomic data on pancreatic cancer from public databases after removing normal tissue specimens and specimens without clinical follow-up data, including the Cancer Genome Atlas (TCGA; https://www.cancer.gov/tcga; *n* = 177) as well as Pancreatic Cancer-Australia (PACA-AU; *n* = 91) and Pancreatic Cancer-Canada (PACA-CA; *n* = 234) from the International Cancer Genome Consortium (ICGC; https://www.icgc-argo.org) (Zhang et al., 2019). Due to samples from different platforms, the batch effects were removed utilizing the ComBat function of the sva package (version 3.42.0) (Leek et al., 2012). A principal component analysis (PCA) was conducted to evaluate the data before and after the removal of the batch effects. The follow-up data and clinicopathological characteristics were also collected. Moreover, single-nucleotide variant (SNV) and copy number variation (CNV) data on pancreatic cancer were retrieved from TCGA project. After reviewing the previously published literature, we collected 159 necroptosis genes, as listed in Supplementary Table S1. The GSE21501 cohort containing expression profiling and follow-up information of 101 pancreatic cancer patients was downloaded from the Gene Expression Omnibus (GEO) repository (https://www.ncbi.nlm.nih.gov/gds/), which was used as the external validation cohort (Stratford et al., 2010; Stratford et al., 2017). Supplementary Figure S1 depicted the workflow of our study.

### Consensus Clustering Analysis

Univariate Cox regression models were conducted between necroptosis genes and pancreatic cancer survival, and genes with *p* < 0.05 were determined for a consensus clustering analysis. A consensus clustering approach offers quantitative and visual stability evidence to estimate the number of unsupervised classes within a specified data set. The ConsensusClusterPlus package (version 1.58.0) adopts the consensus clustering approach, comprising consensus matrix, empirical cumulative distribution function (CDF), and delta area plots (Wilkerson and Hayes, 2010). Through implementing the consensus clustering analysis, necroptosis subtypes were clustered based on the expression values of prognostic necroptosis genes across pancreatic cancer specimens. The number of clusters k was set as 2–9, and 80% of the samples were sampled using a re-sampling method. After multiple sampling, stable and reliable unsupervised classes were found in line with the following parameters: re-samplings = 50, proportion of items to sample = 0.8, proportion of features to sample = 1, and distance = “pearson”. PCA was conducted to visualize the difference in expression levels of prognostic necroptosis genes among diverse necroptosis subtypes.

### Gene Set Variation Analysis

GSVA, a non-parametric and unsupervised gene set enrichment approach, can estimate the enrichment score of specific pathways or signatures in accordance with transcriptomic profiles (Hänzelmann et al., 2013). The 50 hallmarks of gene sets were retrieved from the Molecular Signatures Database (Liberzon et al., 2015). The activity of each hallmark pathway was quantified using the single-sample gene set enrichment analysis (ssGSEA) function.

### Estimation of Tumor Immunogenicity

The relative infiltrations of immune cell populations were estimated with the ssGSEA function derived from the GSVA package (Hänzelmann et al., 2013) on the basis of the expression values of 782 meta-genes (Charoentong et al., 2017) in pancreatic cancer specimens. The mRNA expressions of known immune checkpoints and human leukocyte antigen (HLA) molecules were quantified in each pancreatic cancer specimen.

### Cancer–Immunity Cycle

Chen and Mellman proposed a cancer–immunity cycle to evaluate antitumor immune responses, containing seven steps: 1) release of cancer antigens, 2) cancer antigen presentation, 3) priming and activation, 4) trafficking of T cells to tumors, 5) infiltration of T cells into tumors, 6) recognition of cancer cells by T cells, and 7) killing of cancer cells (Karasaki et al., 2017). The levels of each step within the cancer–immunity cycle were quantified using the ssGSEA approach.

### Quantification of Known Biological Processes

The gene sets of known biological processes were retrieved from Mariathasan et al. (2018), containing epithelial–mesenchymal transition (EMT1-3), immune checkpoint, antigen processing machinery, CD8 T effector, angiogenesis, pan-fibroblast TGFβ response (pan-F-TBRS), DNA damage repair, FGFR3-related genes, KEGG-discovered histones, Fanconi anemia, cell cycle, cell cycle regulators, DNA replication, nucleotide excision repair, homologous recombination, mismatch repair, and WNT target. The enrichment score of aforementioned biological processes was quantified using the ssGSEA approach.

### Chemosensitivity Analysis

The therapeutic responses to known chemotherapy agents (cisplatin, gemcitabine, docetaxel, and paclitaxel) were estimated using the pRRophetic package (Geeleher et al., 2014). Through construction of the ridge regression model on the basis of the Genomics of Drug Sensitivity in Cancer (GDSC) pharmacogenomics database (www.cancerRxgene.org) (Yang et al., 2013) and transcriptomic data, the half-maximal inhibitory concentration (IC50) of each chemotherapeutic agent was calculated across pancreatic cancer specimens.

### Analysis of SNV and CNV Data

Utilizing the maftools package (version 2.10.0) (Mayakonda et al., 2018), SNV data were analyzed and visualized on the basis of the mutation annotation format (MAF) of pancreatic cancer. GISTIC2.0 (Mermel et al., 2011) was implemented to analyze copy number amplification and deletion.

### Identification of Necroptosis-Relevant Genes

A differential expression analysis was implemented between any two necroptosis subtypes utilizing linear models for the microarray data (limma; version 3.50.0) package (Ritchie et al., 2015). Genes with adjusted p-value<0.05 were screened, and necroptosis-relevant genes were determined following the intersection of differentially expressed genes.

### Functional Enrichment Analyses

Utilizing the clusterProfiler package (version 4.2.0) (Yu et al., 2012), a functional enrichment analysis of necroptosis-relevant genes was carried out, comprising Gene Ontology (GO) and Kyoto Encyclopedia of Genes and Genomes (KEGG) pathway enrichment analyses.

### Generation of a Necroptosis-Relevant Risk Model

Prognostic necroptosis–relevant genes with p < 0.05 were determined through univariate Cox regression models, which were ranked by using the randomForestSRC package (version 2.14.0), following number of replication = 100, number of step = 5, Monte Carlo iteration number = 100, and genes with relative importance ˃ 0.4. Thereafter, a necroptosis-relevant risk model was generated on the basis of the expression of the most important genes and regression coefficients from a multivariate Cox regression model. Following calculation of the necroptosis-relevant risk score of each pancreatic cancer patient, high- and low-risk groups were separated in accordance with the median value of risk score. Receiver operator characteristic (ROC) curves at 1-, 3-, and 5-year survival were conducted to evaluate the predictive reliability of the necroptosis-relevant risk model in pancreatic cancer survival. Using the GEPIA web tool (http://gepia.cancer-pku.cn/), the expression of genes in the necroptosis-relevant risk model was examined in pancreatic cancer (*n* = 179) and normal tissues (*n* = 171).

### Construction of a Prognostic Nomogram

In the nomogram, the line length indicates the degree of influence of a specific variable and diverse values of this variable on outcomes. After univariate and multivariate Cox regression models, a nomogram was generated on the basis of independent prognostic factors through the rms package (version 6.2-0), showing the intuitive and effective results of the risk model. Calibration curves were utilized to validate the predictive accuracy of the nomogram-predicted survival probabilities for 1-, 3-, and 5-year survival.

### Statistical Analysis

R software (version 3.6.1) was implemented for data processing. Univariate and multivariate Cox regression analyses were conducted, and the hazards ratio (HR) and p-value were calculated to evaluate the correlations of variables with pancreatic cancer survival. Kaplan–Meier curves and log-rank test were depicted for the survival difference between groups. The difference between two groups was compared with student’s t-test or Wilcoxon test, while comparison between three groups was presented through the Kruskal–Wallis test. A correlation analysis was carried out *via* Pearson’s or Spearman’s test. The C-index was calculated for estimating the prediction performance through the survival package. p < 0.05 indicated statistical significance.

## Results

### Characterization of Five Necroptosis Subtypes With Diverse Survival Outcomes for Pancreatic Cancer

**FIGURE 1:**
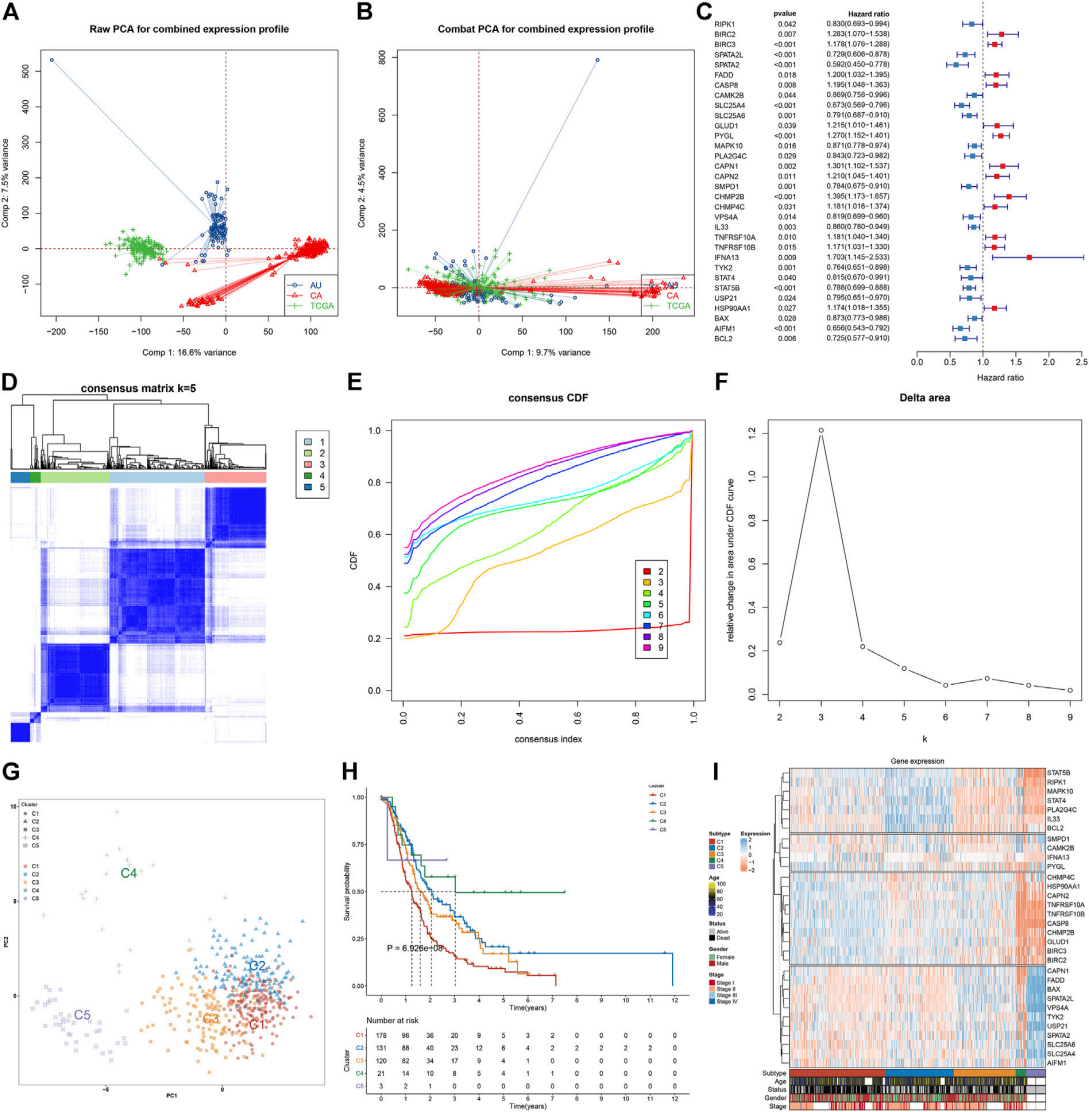
Characterization of five necroptosis subtypes with diverse survival outcomes for pancreatic cancer in the integrated TCGA, PACA-AU, and PACA-CA cohorts. **(A,B)** PCA plots show the data before and after the removal of the batch effects. **(C)** Forest plots visualize the hazard ratios and p-values of prognostic necroptosis genes for pancreatic cancer patients utilizing univariate Cox regression models. Red, risk factor; blue, protective factor. **(D)** Based on the expression values of prognostic necroptosis genes, the consensus matrix is shown when *k* = 5. The rows and columns of the matrix represent samples. The values of the consensus matrix range from 0 (cannot be clustered) to 1 (always clustered) in white to dark blue. **(E)** Consensus CDF plot when k = 2–9. **(F)** Delta area plot when *k* = 2–9. The delta area score (*y*-axis) indicates the relative change in cluster stability. **(G)** PCA plots visualize the difference among five necroptosis subtypes, following the expression values of prognostic necroptosis genes across pancreatic cancer specimens. **(H)** Survival analysis of five necroptosis subtypes. **(I)** Heatmap visualizes the expression of prognostic necroptosis genes in diverse necroptosis subtypes.

We retrospectively collected transcriptomic data on pancreatic cancer from TCGA, PACA-AU, and PACA-CA cohorts. The batch effects of integrated data were eliminated for subsequent analyses, which were visualized through PCA (Figures 1A,B). Among 159 necroptosis genes, 18 genes (*SPATA2*, *AIFM1*, *SLC25A4*, *BCL2*, *SPATA2L*, *TYK2*, *SMPD1*, *STAT5B*, *SLC25A6*, *USP21*, *STAT4*, *VPS4A*, *RIPK1*, *PLA2G4C*, *IL33*, *CAMK2B*, *MAPK10*, and *BAX*) were protective factors of pancreatic cancer prognosis, while 14 genes (*TNFRSF10B*, *HSP90AA1*, *BIRC3*, *TNFRSF10A*, *CHMP4C*, *CASP8*, *FADD*, *CAPN2*, *GLUD1*, *PYGL*, *BIRC2*, *CAPN1*, *CHMP2B*, and *IFNA13*) were risk factors of prognosis, as depicted in Figure 1C. These prognostic necroptosis genes were utilized for the consensus clustering analysis. When *k* = 5, pancreatic cancer samples were clearly separated into five clusters (Figure 1D). Figure 1E depicted the CDF when k takes different values, and we found that when k = 5, CDF reached the approximate maximum, indicative of cluster stability. Figure 1F showed the relative change in CDF of k compared to k-1. When *k* = 6, CDF only slightly decreased, so 5 was the appropriate value of k. Ultimately, five necroptosis subtypes were identified for pancreatic cancer, namely, C1 (*n* = 187), C2 (*n* = 135), C3 (*n* = 121), C4 (*n* = 21), and C5 (*n* = 38). PCA also confirmed the reliability of necroptosis subtypes (Figure 1G). The survival analysis demonstrated the remarkable survival difference among necroptosis subtypes (Figure 1H). The C1 subtype had the worst survival outcomes, followed by C3, C2, C4, and C5. Figure 1I depicted the prominent expression difference of prognostic necroptosis genes among diverse subtypes. The accuracy and reliability of necroptosis subtypes were confirmed in the TCGA cohort (Supplementary Figures S2A-F).

### Necroptosis Subtypes With Diverse Immunogenic Features

**FIGURE 2:**
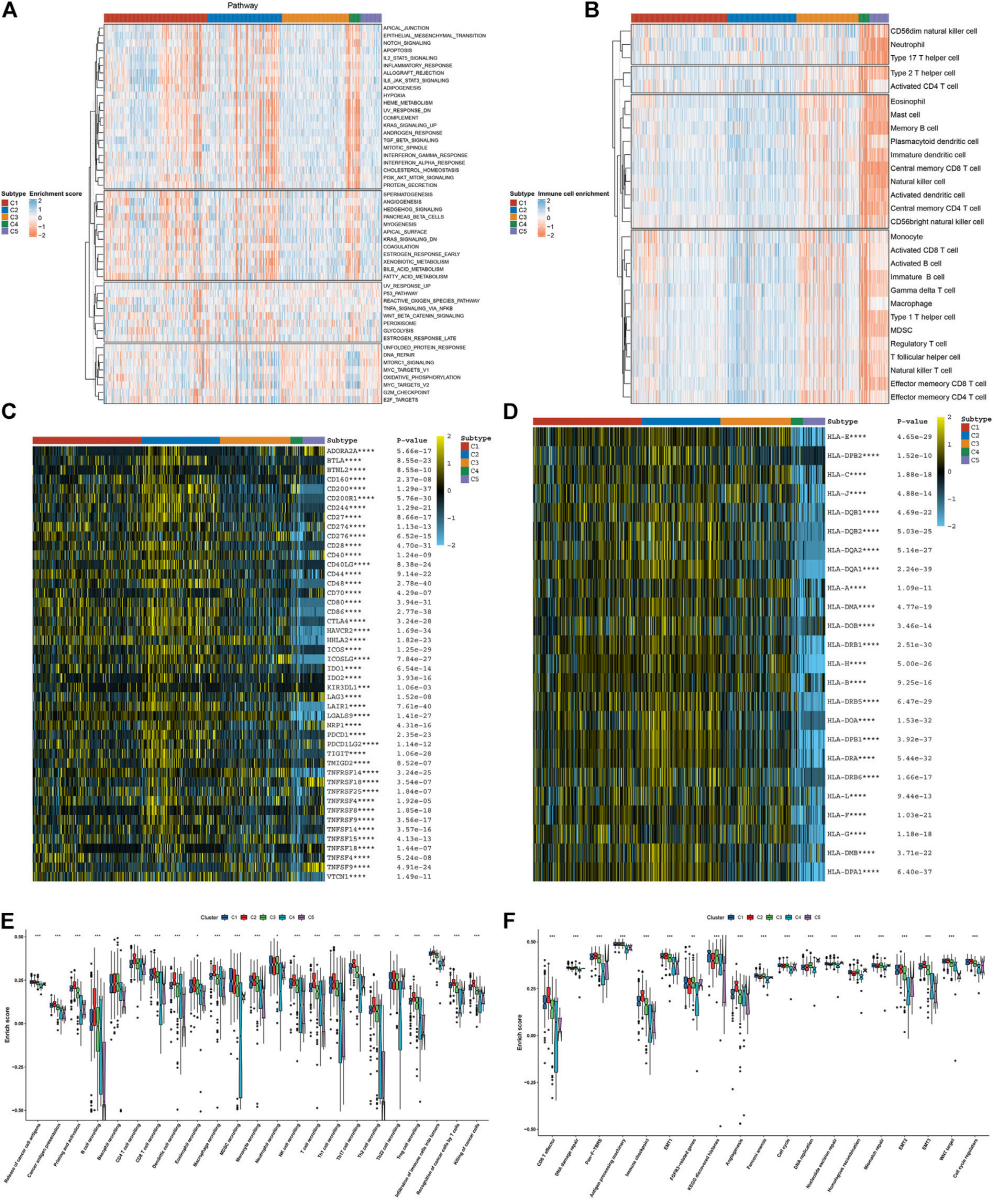
Five necroptosis subtypes with diverse immunogenic features. **(A)** Quantification of the activation levels of known hallmarks of cancer pathways in five necroptosis subtypes. **(B)** Estimation of the infiltration levels of immune cell populations in diverse necroptosis subtypes. **(C,D)** Visualization of the mRNA expression of **(C)** immune checkpoints and **(D)** HLA molecules across necroptosis subtypes. **(E)** Comparison of the enrichment scores of all steps within the cancer–immunity cycle among five necroptosis subtypes. **(F)** Comparison of the enrichment scores of known biological processes in five necroptosis subtypes. *p < 0.05; **p < 0.01; ***p < 0.001; and ****p < 0.0001.

Further analysis was conducted to uncover the mechanisms underlying five necroptosis subtypes. In Figure 2A, tumorigenic pathways (hedgehog signaling, KRAS, angiogenesis, glycolysis, etc.) were remarkably activated in C1 and C2 subtypes, contributing to an undesirable prognosis. C4 and C5 subtypes presented the relatively high infiltrations of immune cells, while C2 was characterized by low infiltrations of immune cells (Figure 2B). Most immune checkpoints were markedly downregulated in C4 and C5 subtypes, while their upregulations were found in C2 (Figure 2C). Tumors can evade T-cell responses through losing the major histocompatibility complex (MHC)/HLA class I and II molecules (Godfrey et al., 2018). In Figure 2D, we observed the loss of HLA class I and II molecules in C4 and C5. Differently, C2 displayed the prominent activation of HLA molecules, followed by relatively modest expression in C1 and C3. C4 and C5 subtypes presented the relatively low levels of almost all steps within the cancer–immunity cycle in comparison to other subtypes; meanwhile, the C2 subtype had the highest activation of each step (Figure 2E). Similarly, CD8 T effector and antigen processing machinery, immune checkpoint, and stromal activation (EMT1-3) were relatively downregulated in C4 and C5 (Figure 2F); C2 had relatively high levels of immune and stromal activation pathways; and C1–3 presented the enhanced cell cycle progression (cell cycle, cell cycle regulators, DNA replication, etc.). Overall, five necroptosis subtypes had diverse immunogenic features.

### Necroptosis Subtypes With Different Chemosensitivity and Tumor Mutation Features

**FIGURE 3:**
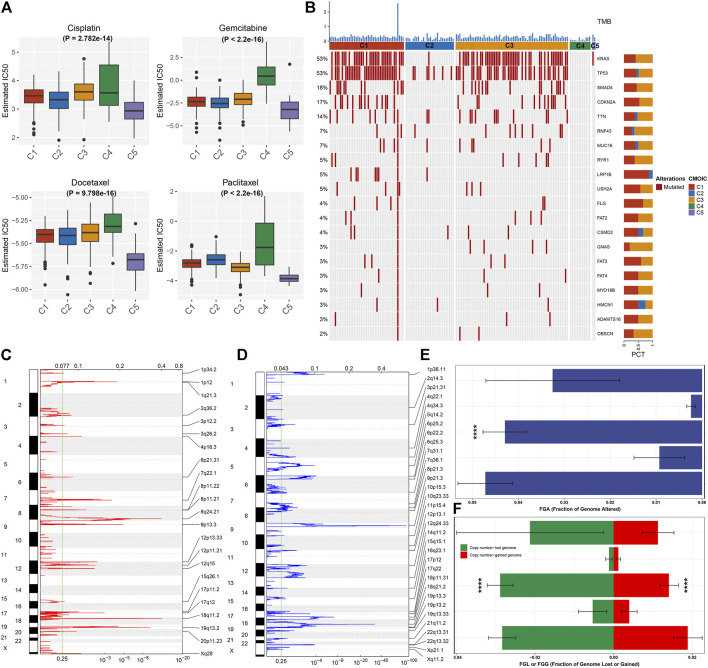
Necroptosis subtypes with different chemosensitivity and tumor mutation features. **(A)** Comparison of the IC50 values of chemotherapy agents (cisplatin, gemcitabine, docetaxel, and paclitaxel) among five necroptosis subtypes. **(B)** Waterfall diagram depicts the first 20 mutated genes and TMB (upper of the panel) across necroptosis subtypes. **(C,D)** Chromosome arms with significant amplification and deletion (q-value <0.25). The focal peak of amplification and deletion is separately visualized. Red indicates copy number amplification, while blue represents copy number deletion. **(E)** Comparison of the fraction of genome altered (FGA) among necroptosis subtypes. **(F)** Comparison of the copy number amplification (red) and deletion (green) in five necroptosis subtypes. ****p < 0.0001.

We compared the sensitivity to known chemotherapy agents (cisplatin, gemcitabine, docetaxel, and paclitaxel) in five necroptosis subtypes. As depicted in Figure 3A, C4 had the highest IC50 values of cisplatin, gemcitabine, docetaxel, and paclitaxel, while C5 presented the lowest IC50 values of aforementioned chemotherapy agents, indicating that C4 presented the highest probability of chemotherapy resistance while C5 was the most sensitive to these chemotherapy agents. We also investigated that C1 and C3 had relatively higher tumor mutation burden (TMB) than other subtypes (Figure 3B). KRAS (53%) and TP53 (53%) were the most frequent mutant genes. The widespread copy number amplification (Figure 3C) and deletion (Figure 3D) occurred in pancreatic cancer specimens. Among five necroptosis subtypes, C2 and C4 had the relatively decreased fractions of genome altered (FGAs), as depicted in Figure 3E. Moreover, we noted the relatively lowered copy number amplification and deletion in C2 and C4 in comparison to other subtypes (Figure 3F). Aforementioned data uncovered the difference in tumor mutations among necroptosis subtypes.

### Generation of a Necroptosis-Relevant Risk Model for Pancreatic Cancer Prognosis

**FIGURE 4:**
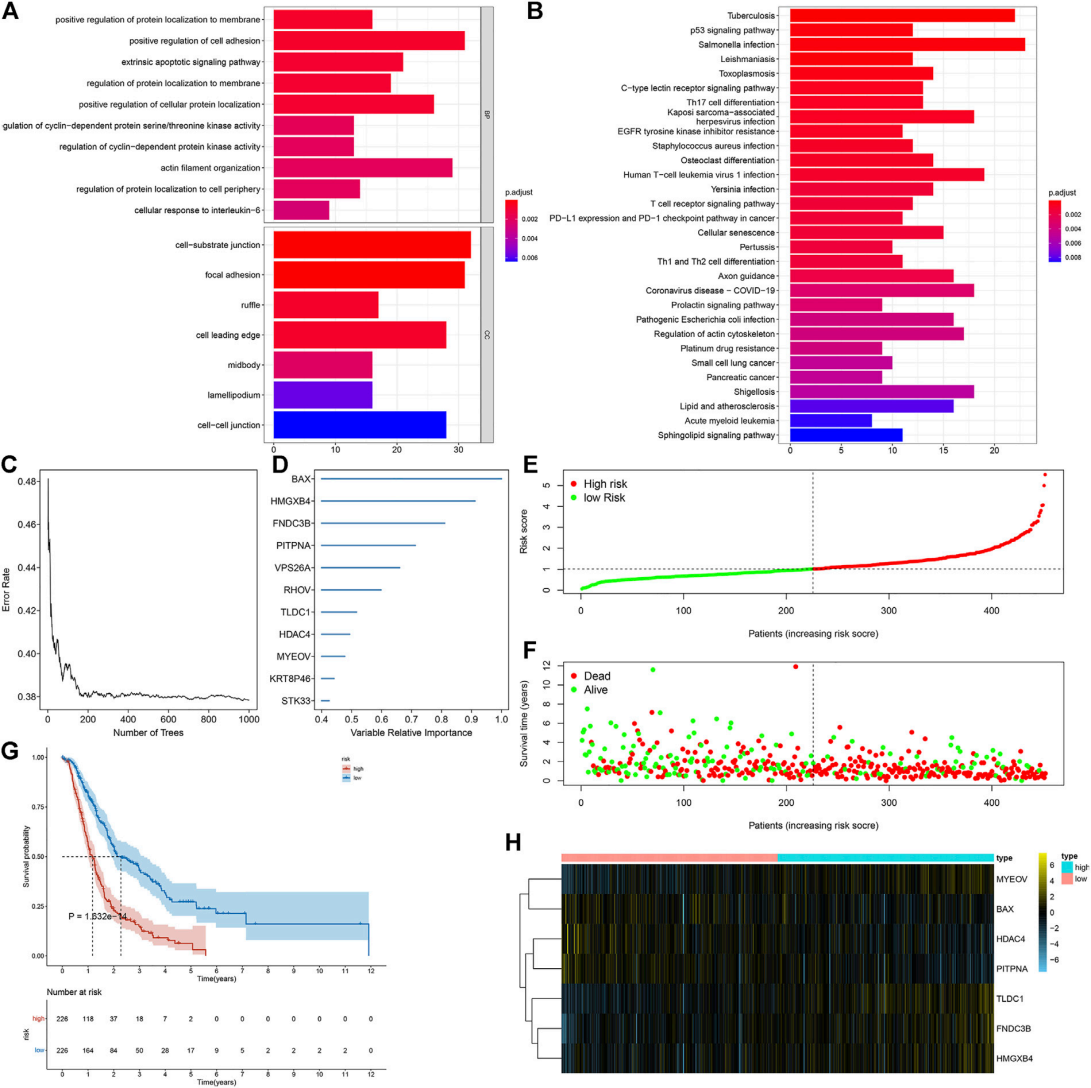
Generation of a necroptosis-relevant risk model for pancreatic cancer prognosis. **(A,B)** GO and KEGG enrichment results of necroptosis-relevant genes. **(C,D)** Most important necroptosis-relevant genes ordered by the relative importance through the random forest approach. **(E,F)** Distribution of the risk score and survival status in high- and low-risk groups. **(G)** Survival analysis of high- and low-risk groups. **(H)** Heatmap depicts the expression of genes in the necroptosis-relevant risk model between two groups.

Through the intersection of DEGs (adjusted *p* < 0.05) between any two subtypes, we determined 591 necroptosis-relevant genes (Supplementary Table S2). Their biological significance was further analyzed through GO and KEGG enrichment analyses. In Figure 4A, necroptosis-relevant genes were remarkably linked with regulation of protein localization to the membrane. Moreover, they had prominent associations with tumorigenic pathways (p53 signaling pathway and cell senescence) and immune pathways (Th17 cell differentiation, PD-L1 expression, PD-1 checkpoint pathway in cancer, Th1 and Th2 cell differentiation, etc.), indicative of the critical roles of necroptosis-relevant genes in pancreatic cancer progression (Figure 4B). Among all necroptosis-relevant genes, 207 displayed significant correlations to pancreatic cancer prognosis (Supplementary Table S3). Using the random forest approach, we determined the most important genes with the relative importance > 0.4 (Figures 4C,D). A multivariate Cox regression model was constructed in line with the following formula: risk score = 0.119399555 * MYEOV expression + (−0.258345687) * HDAC4 expression + 0.26238863 * TLDC1 expression + (−0.395042137) * PITPNA +0.175544976 * FNDC3B expression + 0.338675676 * HMGXB4 expression + (−0.150557275) * BAX expression. Following the calculation of the risk score, all patients were separated into high- and low-risk groups (Figure 4E). The high-risk group had more dead patients relative to the low-risk group (Figure 4F). The survival analysis demonstrated the survival advantage of low-risk patients (Figure 4G). The difference in expression of aforementioned genes between groups is visualized in Figure 4H.

### Necroptosis-Relevant Risk Model as a Reliable and Independent Prognostic Indicator of Pancreatic Cancer

**FIGURE 5:**
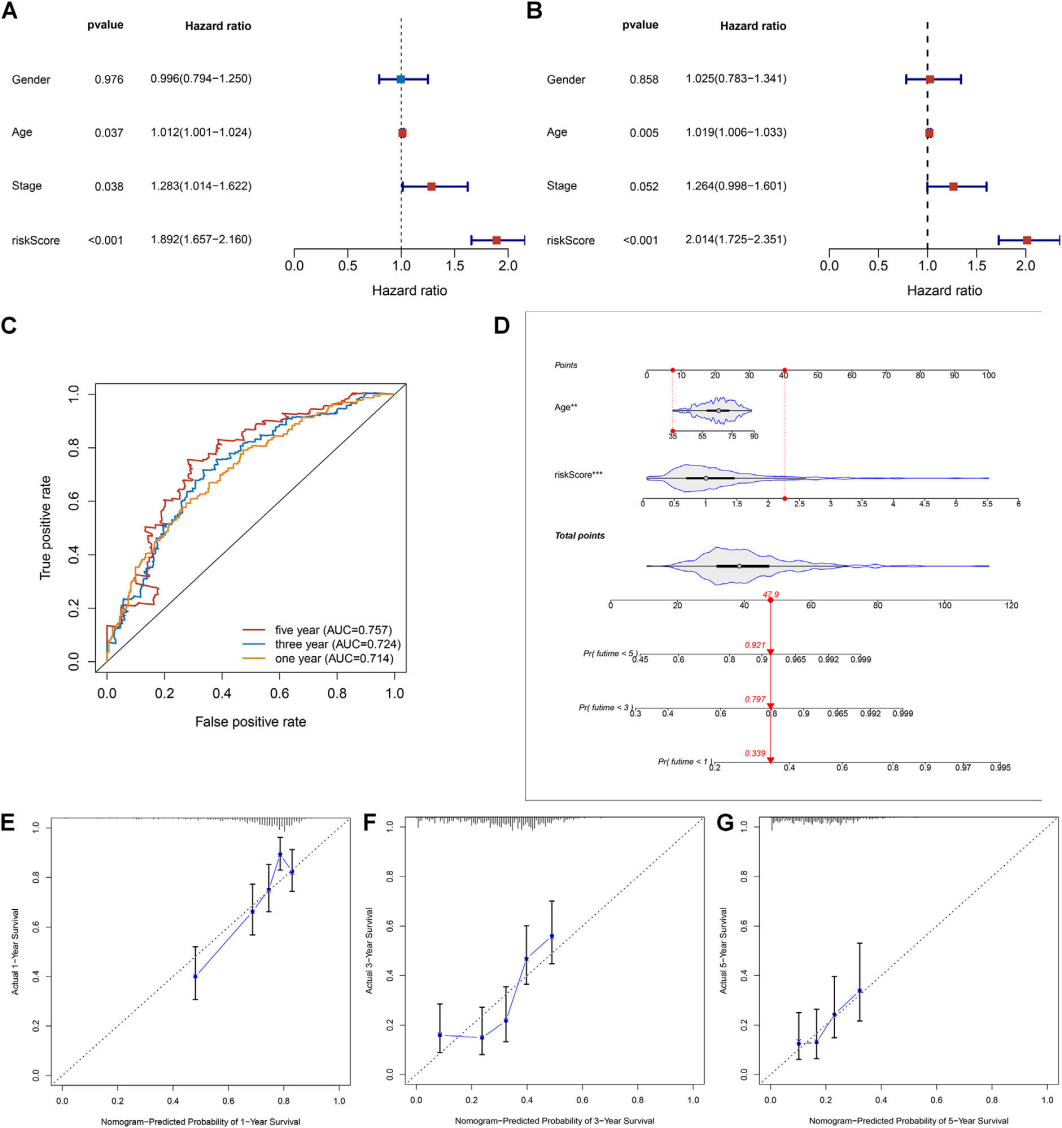
Necroptosis-relevant risk model as a reliable and independent prognostic indicator of pancreatic cancer. **(A,B)** Forest plots show the correlations of the necroptosis-relevant risk score, age, and stage with pancreatic cancer prognosis through **(A)** uni- and **(B)** multivariate Cox regression models. **(C)** ROC curves at 1-, 3-, and 5-year survival for the necroptosis-relevant risk score. **(D)** Generation of an age- and risk score–based prognostic nomogram. **(E–G)** Calibration curves depict the deviations between nomogram-predicted probabilities of 1-, 3-, and 5-year survival and actual survival outcomes.

Uni- and multivariate Cox regression analyses uncovered that age- and necroptosis-relevant risk models were both independently associated with pancreatic cancer survival (Figures 5A,B). AUCs at 1-, 3-, and 5-year survival were separately 0.714, 0.724, and 0.757, indicative of the reliability of the necroptosis-relevant risk model in predicting survival outcomes (Figure 5C). To facilitate the clinical application of the necroptosis-relevant risk model, we generated a nomogram following integration of age (Figure 5D). Calibration curves demonstrated the predictive accuracy of this nomogram in pancreatic cancer survival (Figures 5E–G). In addition, we also performed a stratified analysis and demonstrated that the risk model can serve as an independent prognostic factor without consideration of the impact of age (Supplementary Figures S3A,B).

### Externally Verifying the Necroptosis-Relevant Risk Model

**FIGURE 6:**
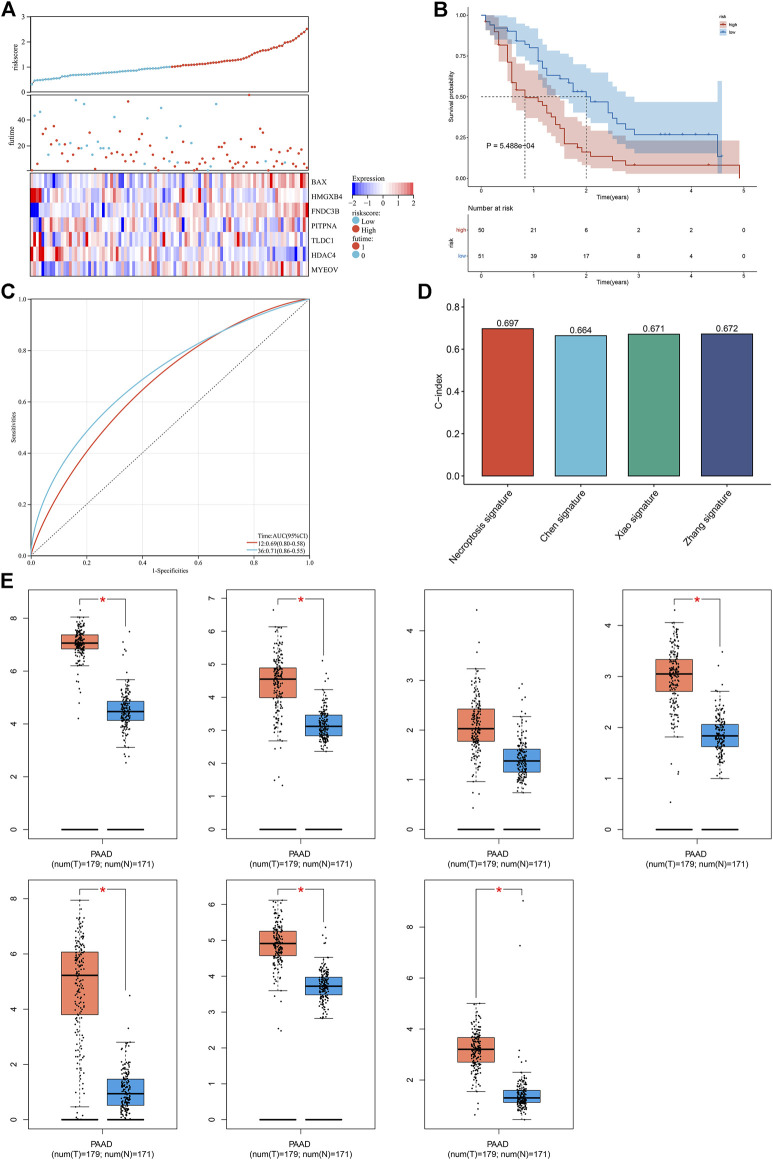
External verification of the necroptosis-relevant risk model. **(A)** Distribution of the necroptosis-relevant risk score, survival status, and expression of necroptosis-relevant genes in the GSE21501 cohort. **(B,C)** Survival analysis and ROC curves in the GSE21501 cohort. **(D)** Comparison of the C-index of the necroptosis-relevant risk score with known prognostic signatures. **(E)** Box plots of the expression of necroptosis-relevant genes using the GEPIA web tool. *p < 0.05.

The robustness of the necroptosis-relevant risk model was verified in the GSE21501 cohort. In accordance with the same formula, we computed the necroptosis-relevant risk score of each pancreatic cancer patient in the external cohort (Figure 6A). As expected, high-risk patients had poorer survival outcomes than low-risk patients (Figure 6B). AUCs at 1- and 3-year survival were separately 0.69 and 0.71 (Figure 6C), demonstrating the excellent performance in predicting prognosis. Compared with the existing prognostic models constructed by Chen et al. (2021), Xiao et al. (2022), and Zhang et al. (2022), the necroptosis-relevant risk model had a higher C-index (Figure 6D), indicating the advantage of this model in predicting prognosis. We also examined the expression of genes in the necroptosis-relevant risk model using the GEPIA web tool. BAX, FNDC3B, HDAC4, HMGXB4, MYEOV, and TLDC1 displayed upregulated expressions in pancreatic cancer than normal tissues (Figure 6E).

### Necroptosis-Relevant Risk Model Correlates With Tumor Immunogenicity for Pancreatic Cancer

**FIGURE 7:**
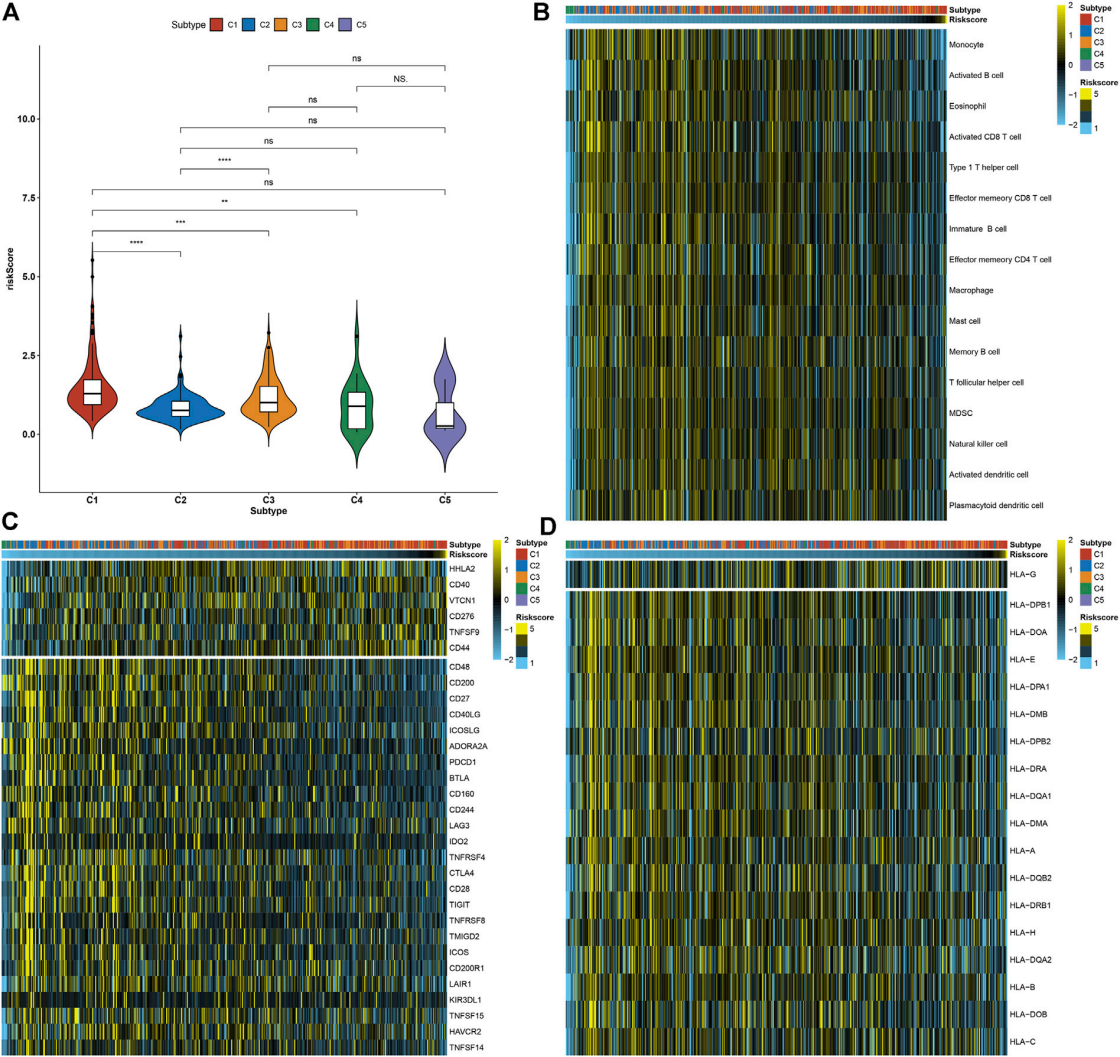
Necroptosis-relevant risk model links with tumor immunogenicity for pancreatic cancer. **(A)** Distribution of the necroptosis-relevant risk score in five necroptosis subtypes. Ns: not significant; **p < 0.01; ***p < 0.001; and ****p < 0.0001. **(B)** Visualization of the infiltrations of immune cell populations in pancreatic cancer specimens ordered by the necroptosis-relevant risk score. **(C,D)** Quantification of the mRNA expression of **(C)** immune checkpoints and **(D)** HLA molecules in pancreatic cancer specimens ordered by the necroptosis-relevant risk score.

Compared with other necroptosis subtypes, C1 presented a relatively higher necroptosis-relevant risk score, followed by C3 (Figure 7A), indicating the heterogeneity in the risk score among diverse necroptosis subtypes. Further analysis was conducted to evaluate the correlations of necroptosis-relevant risk score with tumor immunogenicity. In Figure 7B, as the necroptosis-relevant risk score increased, the infiltrations of immune cells gradually decreased, indicative of the negative correlations of the necroptosis-relevant risk score with immune cell infiltrations. Moreover, we noted that the necroptosis-relevant risk score was negatively linked with the expression of immune checkpoints and HLA molecules (Figures 7C,D). The aforementioned data indicated the role of the necroptosis-relevant risk model in tumor immunogenicity of pancreatic cancer.

### Necroptosis-Relevant Risk Model Links With the Cancer Immunity Cycle and Known Biological Processes

**FIGURE 8:**
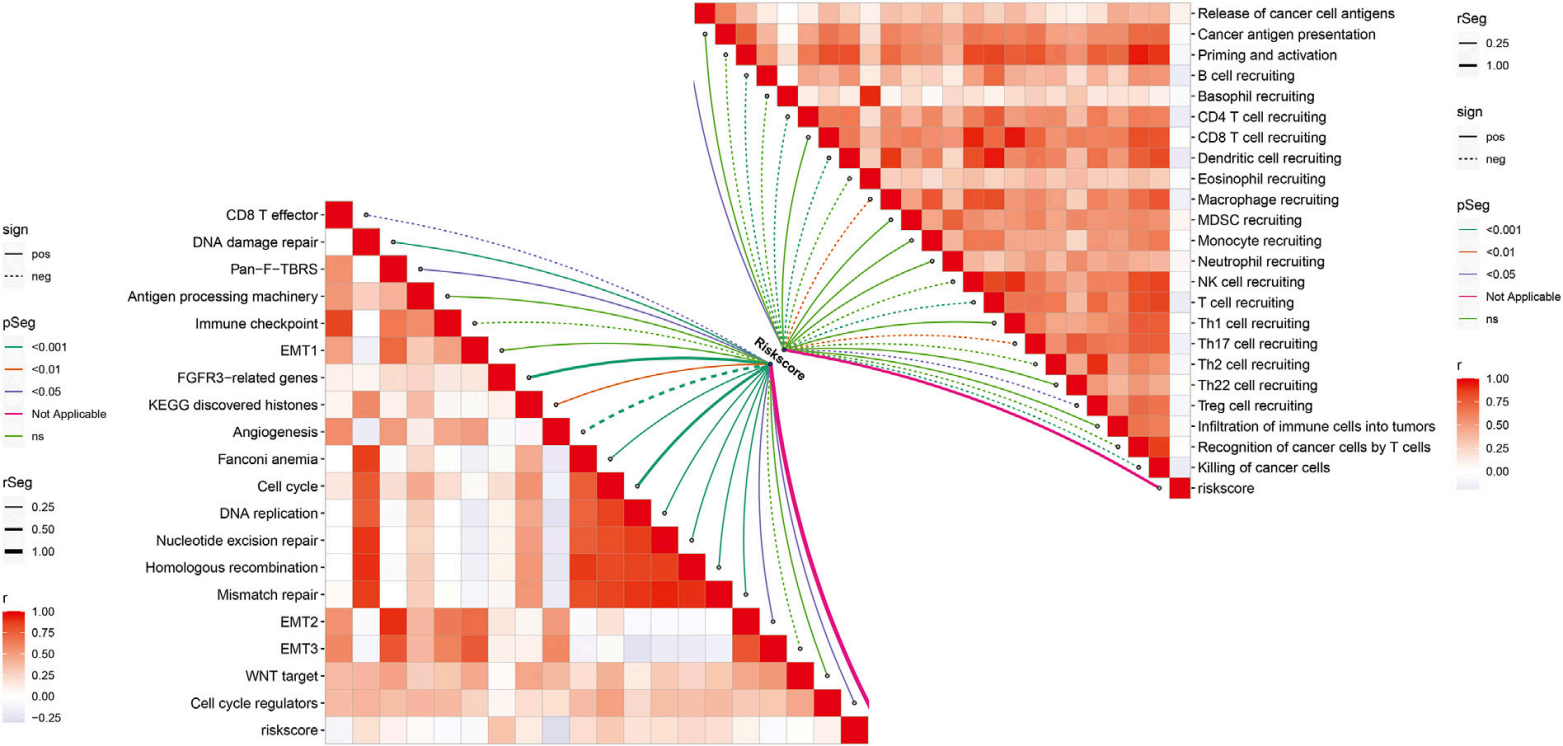
Necroptosis-relevant risk model links with the cancer–immunity cycle and known biological processes in pancreatic cancer. Spearman’s correlation analysis was conducted between the necroptosis-relevant risk score and all steps within the cancer–immunity cycle and known biological processes.

In Figure 8, we noted that the necroptosis-relevant risk score presented a significantly positive correlation to the release of cancer cell antigens but displayed significantly negative correlations to recruiting of B cells, CD4 T cells, dendritic cell, macrophages, T cells, Th17 cells, Treg cells, and killing of cancer cells, indicative of the remarkable interactions of the necroptosis-relevant risk score with the cancer–immunity cycle. Moreover, necroptosis-relevant risk score was negatively linked with CD8 T effector, and angiogenesis but was positively associated with pan-F-TBRS, FGFR3-related genes, EMT2, KEGG discovered histones, Fanconi anemia, cell cycle, cell cycle regulators, DNA replication, DNA damage repair, nucleotide excision repair, homologous recombination, and mismatch repair, indicative of the mechanisms underlying the necroptosis-relevant risk score.

### Necroptosis-Relevant Risk Model Correlates With Chemosensitivity of Pancreatic Cancer

**FIGURE 9:**
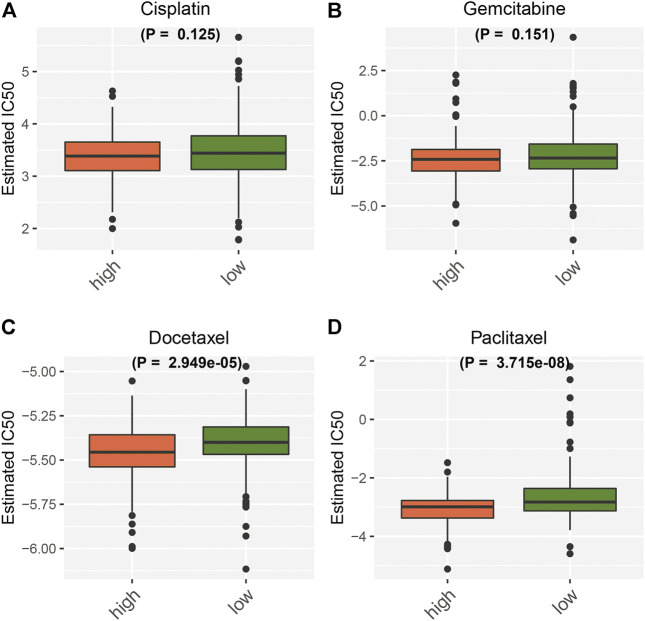
Necroptosis-relevant risk model correlates with the chemosensitivity of pancreatic cancer. Box plots show the IC50 values of **(A)** cisplatin, **(B)** gemcitabine, **(C)** docetaxel, and **(D)** paclitaxel in high- and low-risk groups.

Further analysis was conducted to evaluate the correlations between the necroptosis-relevant risk score and chemosensitivity of pancreatic cancer. No remarkable differences of the IC50 values of cisplatin and gemcitabine were noticed between high- and low-risk groups (Figures 9A,B). But the high-risk group presented the prominently reduced IC50 values of docetaxel and paclitaxel relative to the low-risk group (Figures 9C,D). This indicated that high-risk patients were more likely to be sensitive to docetaxel and paclitaxel.

## Discussion

Since there are currently no specific molecular biomarkers for detecting necroptosis, identifying necroptosis usually requires combined detection approaches (Niu et al., 2022). Under transmission electron microscopy, necrotic morphology is identified. Detecting necroptosis by biomarkers mainly focuses on the pivotal molecular events involving necroptosis. Nevertheless, the exact roles of necroptosis in pancreatic cancer remain to be adequately clarified. Herein, we proposed five necroptosis-based molecular subtypes and a necroptosis-relevant risk model for pancreatic cancer through integrated analysis of necroptosis genes, which expanded the understanding of necroptosis in pancreatic cancer biology.

Consensus clustering analysis is beneficial to provide patients with more accurate therapy, referring to a situation where diverse clusters are acquired for a specific dataset and desired for aggregating the clustering results to obtain an in-depth clustering solution (Li et al., 2020). On the basis of the expression values of prognostic necroptosis genes, five necroptosis subtypes were determined for pancreatic cancer, with diverse survival outcomes. C1 subtype had the worst survival outcomes, followed by C3, C2, C4 and C5. Tumorigenic pathways (hedgehog signaling, KRAS, angiogenesis, glycolysis, etc.) were remarkably activated in C1 and C2 subtypes, contributing to unfavorable survival outcomes. The five subtypes presented diverse expression patterns of necroptosis genes. Elucidating the exact regulatory mechanisms of necroptosis can facilitate the development of new therapeutic strategies to overcome apoptosis resistance in pancreatic cancer. Tumors express various MHC molecules, which can be targets for specific cytotoxic T lymphocytes, resulting in them to be immunogenic. Most immune checkpoints and HLA molecules were markedly downregulated in C4 and C5 subtypes while their upregulations were found in the C2 subtype. This indicated that tumors in C4 and C5 with robust T-cell immunosurveillance presented disable antigen presentation to evade immunorecognition. The cancer–immunity cycle contains seven stepwise steps for obtaining an efficient control of tumor growth through the immune system, which is initiated *via* the release of neo-antigens produced by genomic instability (Huntington et al., 2020). Cancer-associated antigens are captured by dendritic cells, and after dendritic cells migrate to lymph nodes, they trigger and activate tumor-specific cytolytic CD8^+^ T cells. These effector cells migrate and penetrate the tumor stroma, where they are able to recognize and kill tumor cells. The cytotoxic response mediated by T cells releases new tumor antigens and promotes the cancer immune cycle. In comparison to other subtypes, C4 and C5 had the relatively rate-limiting steps within the cancer immune cycle. Hence, five subtypes possessed diverse immunogenic features and were predictive of immunotherapeutic responses.

Accumulated evidence suggests the links of necroptosis with chemotherapy resistance (Gong et al., 2019). Cisplatin is a crucial agent for treatment of pancreatic cancer patients with BRCA1/2 or PALB2 mutation (Kong et al., 2020). Apoptosis resistance represents a primary obstacle resulting in chemotherapy failure. Bypassing the apoptotic pathway to induce cancer cell death is a promising therapeutic option to overcome this issue (Gong et al., 2019). Necroptosis is an alternative mode of programmed cell death to overcome apoptosis resistance. Experimental evidence shows that cisplatin induces necroptosis with tumor necrosis factor *α* (TNFα)-dependent and independent pathways (Xu et al., 2017). Moreover, multitargeted kinase inhibitor KW-2449 alleviates cisplatin-induced nephrotoxicity through targeting RIPK1-independent necroptosis (Rui et al., 2021). Combination therapy of CD95L and gemcitabine facilitates RIP1-independent necroptosis in pancreatic cancer cells (Pietkiewicz et al., 2015). Necroptosis can alleviate docetaxel resistance in prostate cancer (Markowitsch et al., 2021) and breast cancer (Mann et al., 2020). Five necroptosis-based molecular subtypes presented diverse sensitivity to chemotherapeutic agents (cisplatin, gemcitabine, docetaxel, and paclitaxel). Among them, the C4 subtype presented the highest probability of chemotherapy resistance while C5 subtype was most sensitive to these chemotherapy agents, indicating that patients with the C5 subtype were most likely to benefit from chemotherapy. Moreover, the necroptosis-relevant risk score can predict the sensitivity to docetaxel and paclitaxel in pancreatic cancer.

A necroptosis-relevant risk model was developed for predicting pancreatic cancer survival and responses to immuno- and chemotherapy, comprising MYEOV, HDAC4, TLDC1, PITPNA, FNDC3B, HMGXB4, and BAX. External validation confirmed that this model was capable of accurately predicting pancreatic cancer patients’ survival outcomes. The genes in the necroptosis-relevant risk model were upregulated in pancreatic cancer in comparison to normal tissues. Previous research has uncovered the significance of aforementioned genes in pancreatic cancer progression. For instance, MYEOV upregulation is linked with undesirable survival outcome of pancreatic cancer (Tang et al., 2020a), which elevates the HES1 expression and triggers pancreatic cancer progression through enhancing SOX9 trans-activity (Liang et al., 2020). HDAC4 correlates with the proliferative capacity and metastases of pancreatic cancer (Cohen et al., 2013). TLDC1 can facilitate proliferation and migration of pancreatic cancer cells (Yuan et al., 2021).

Several limitations should be pointed out in this study. First of all, clinical information retrieved from TCGA and ICGC projects is not complete, especially the therapy, which may assist in comprehending whether necroptosis genes are biomarkers of therapeutic responses. Second, the mechanisms how necroptosis modulate the precise process of pancreatic cancer are indistinct. Third, the necroptosis-relevant risk model is required to verify in large-scale and multicenter clinical cohorts. Despite these limitations, this study does offer a comprehensive overview of necroptosis gene profiling in pancreatic cancer, and aforementioned limitations will be solved if there are sufficient data in our further research.

## Conclusion

Collectively, we characterized five robust necroptosis subtypes for pancreatic cancer with diverse prognosis, immunogenic features, genomic mutations, and chemosensitivity. Furthermore, we established a necroptosis-relevant risk model for reflecting necroptosis in clinical practice. Our findings offered a reference for combined therapeutic regimens and might guide the optimal selection of patients for immuno- and chemotherapy.

## Data Availability

The original contributions presented in the study are included in the article/Supplementary Material, further inquiries can be directed to the corresponding author.

## Abbreviations

CDF, cumulative distribution function; CNV, copy number variation; EMT, epithelial–mesenchymal transition; GDSC, Genomics of Drug Sensitivity in Cancer; GO, Gene Ontology; GSVA, gene set variation analysis; HLA, human leukocyte antigen; HR, hazards ratio; ICGC, International Cancer Genome Consortium; IC50, half-maximal inhibitory concentration; KEGG, Kyoto Encyclopedia of Genes and Genomes; limma, linear models for microarray data; MAF, mutation annotation format; MLKL, mixed lineage kinase domain-like; PACA-AU, Pancreatic Cancer-Australia; PACA-CA, Pancreatic Cancer-Canada; pan-F-TBRS, pan-fibroblast TGFβ response; PCA, principal component analysis; ROC, receiver operator characteristic; RIPK1, receptor-interacting protein kinase 1; SNV, single-nucleotide variant; ssGSEA, single-sample gene set enrichment analysis; TCGA, The Cancer Genome Atlas; TRIM28, tripartite motif protein 28.
