# Supplementary figures and images for: Comprehensive Analysis of Necroptosis in Pancreatic Cancer for Appealing its Implications in Prognosis, Immunotherapy, and Chemotherapy Responses
Source: Front Pharmacol. 2022 May 18;13:862502. doi: 10.3389/fphar.2022.862502 (PMC9157651; doi:10.3389/fphar.2022.862502)

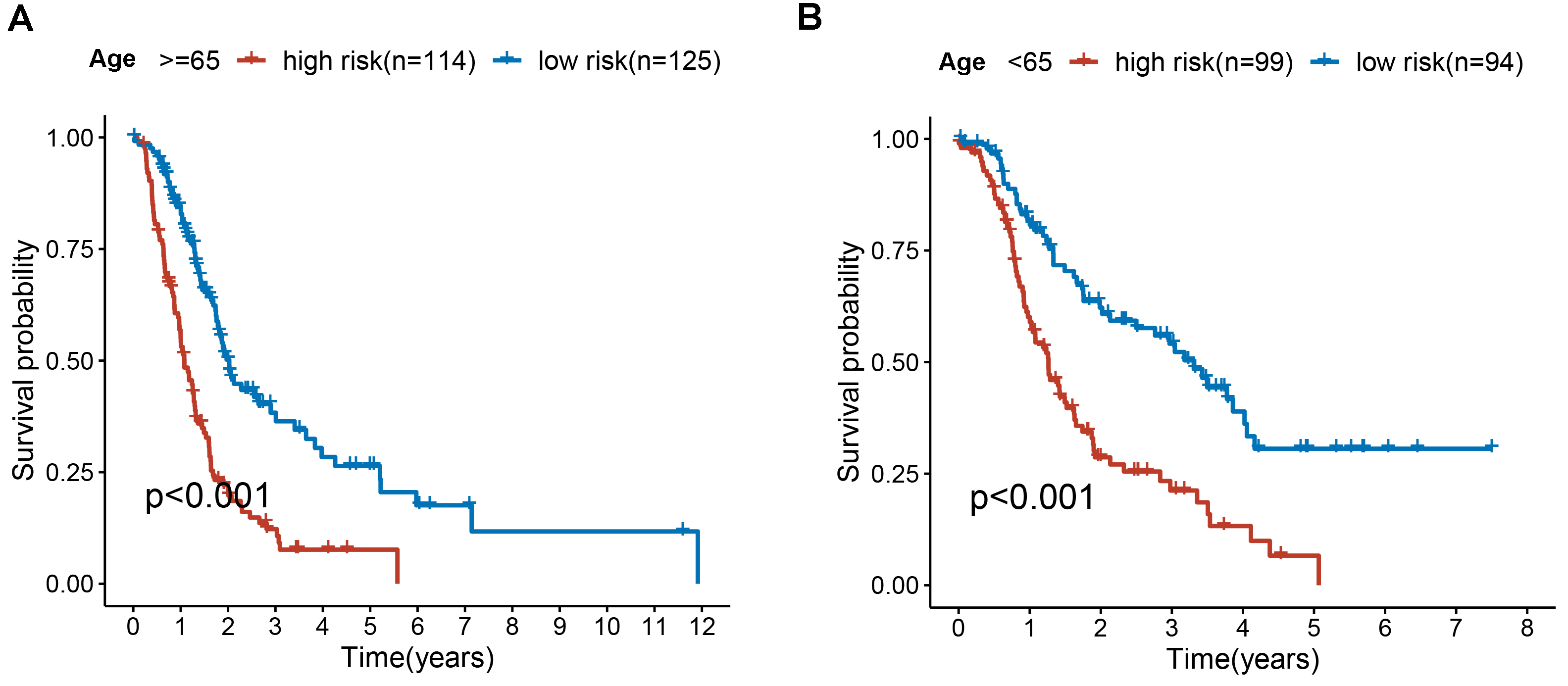

Supplement: Supplementary file 3 [file Image3.TIF]

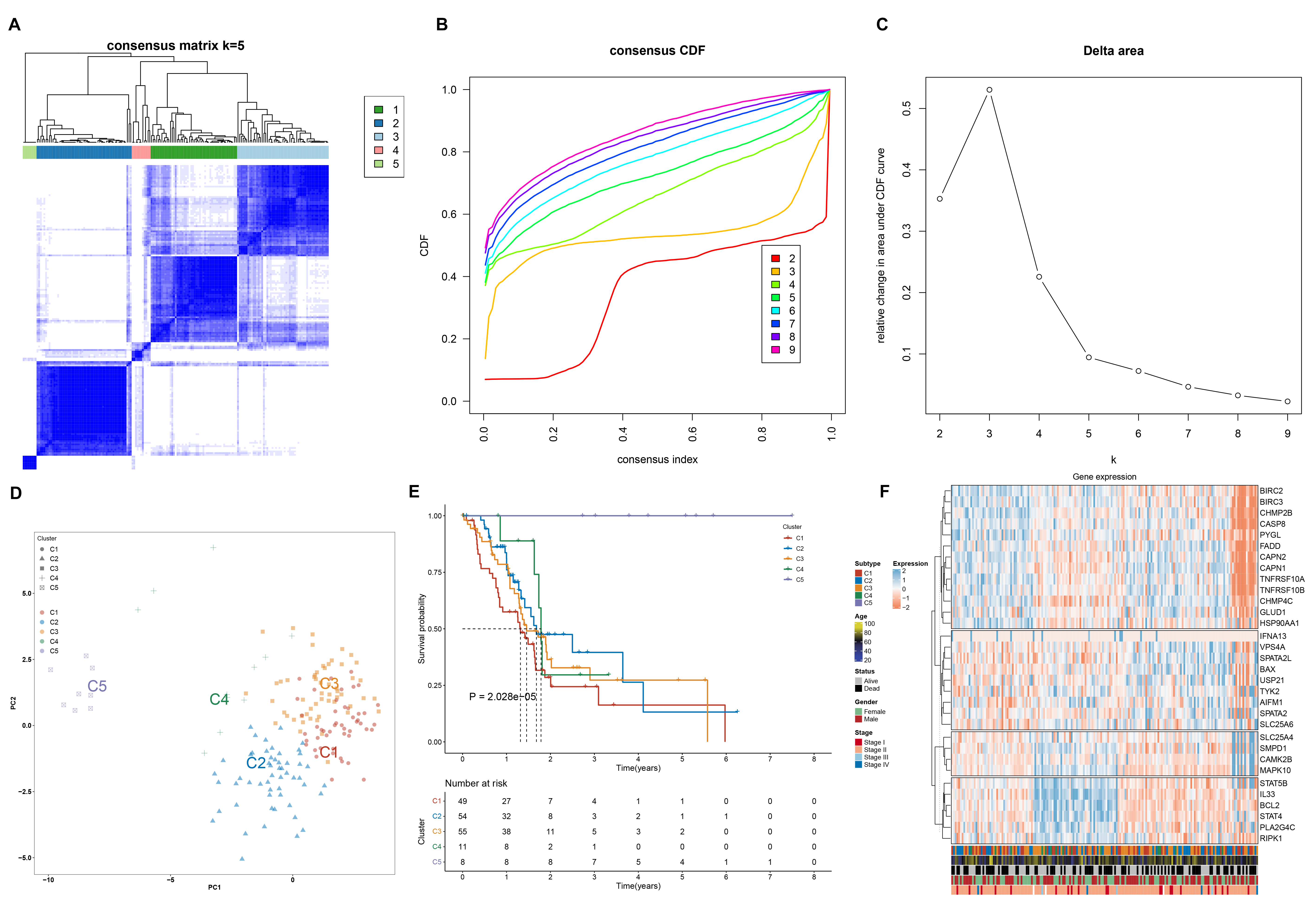

Supplement: Supplementary file 4 [file Image2.TIF]

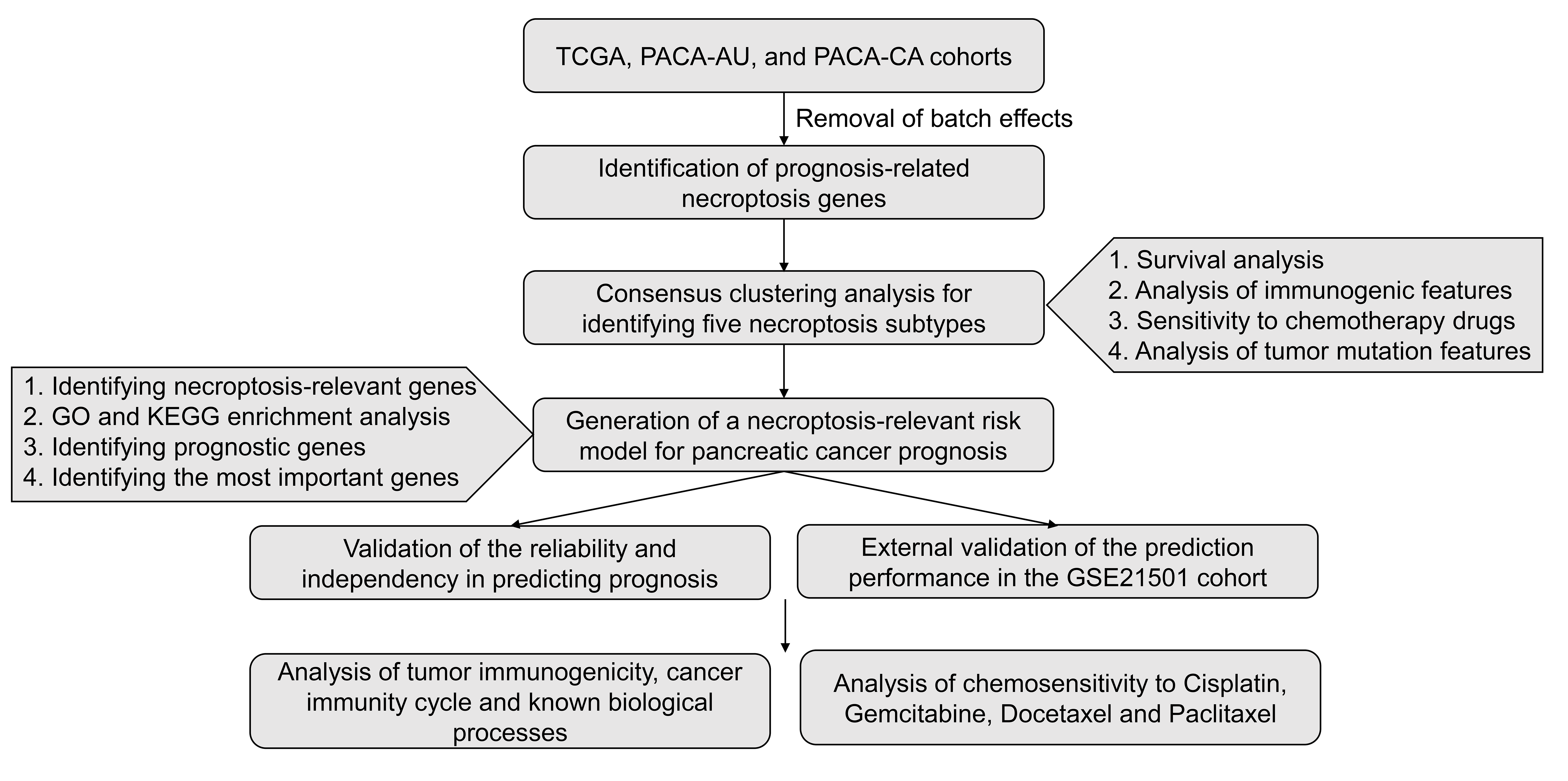

Supplement: Supplementary file 5 [file Image1.TIF]
